# Clinical and preclinical evidence that angiotensin-converting enzyme inhibitors and angiotensin receptor blockers prevent diabetic peripheral neuropathy

**DOI:** 10.1038/s41598-024-51572-z

**Published:** 2024-01-10

**Authors:** Shiori Iwane, Wataru Nemoto, Tomoyoshi Miyamoto, Tomonori Hayashi, Masayuki Tanaka, Kazuki Uchitani, Tatsuya Muranaka, Masanori Fujitani, Yuichi Koizumi, Atsushi Hirata, Maho Tsubota, Fumiko Sekiguchi, Koichi Tan-No, Atsufumi Kawabata

**Affiliations:** 1 Laboratory of Pharmacology and Pathophysiology, Faculty of Pharmacy, Kindai University, Higashi-Osaka, 577-8502 Japan; 2 Department of Pharmacy, Kansai Medical University Hospital, Hirakata, 573-1191 Japan; 3 Division of Pharmacology, Faculty of Pharmaceutical Sciences, Tohoku Medical and Pharmaceutical University, Sendai, 981-8558 Japan; 4 School of Pharmacy, Hyogo Medical University, Kobe, 650-8530 Japan; 5 Department of Pharmacy, Kindai University Nara Hospital, Ikoma, 630-0293 Japan; 6 Department of Pharmacy, Seichokai Fuchu Hospital, Izumi, 594-0076 Japan

**Keywords:** Sensory processing, Neuroscience, Endocrinology

## Abstract

Given possible involvement of the central and peripheral angiotensin system in pain processing, we conducted clinical and preclinical studies to test whether pharmacological inhibition of the angiotensin system would prevent diabetic peripheral neuropathy (DPN) accompanying type 2 diabetes mellitus (T2DM). In the preclinical study, the nociceptive sensitivity was determined in leptin-deficient *ob*/*ob* mice, a T2DM model. A clinical retrospective cohort study was conducted, using the medical records of T2DM patients receiving antihypertensives at three hospitals for nearly a decade. In the *ob*/*ob* mice, daily treatment with perindopril, an angiotensin-converting enzyme inhibitor (ACEI), or telmisartan, an angiotensin receptor blocker (ARB), but not amlodipine, an L-type calcium channel blocker (CaB), significantly inhibited DPN development without affecting the hyperglycemia. In the clinical study, the enrolled 7464 patients were divided into three groups receiving ACEIs, ARBs and the others (non-ACEI, non-ARB antihypertensives). Bonferroni’s test indicated significantly later DPN development in the ARB and ACEI groups than the others group. The multivariate Cox proportional analysis detected significant negative association of the prescription of ACEIs or ARBs and β-blockers, but not CaBs or diuretics, with DPN development. Thus, our study suggests that pharmacological inhibition of the angiotensin system is beneficial to prevent DPN accompanying T2DM.

## Introduction

Type 2 diabetes mellitus (T2DM) and hypertension are closely related with metabolic syndrome, and interact synergistically to promote cardiovascular diseases and kidney dysfunction^1,2^. The use of the renin-angiotensin (Ang) system (RAS)-blocking agents for blood pressure control is recommended to prevent the development of chronic kidney disease and cardiovascular events in DM patients^3^. Diabetic peripheral neuropathy (DPN) is one of the most frequent complications in DM patients, and the incidence of DPN is approximately 31.5% and 17.5% in T2DM and T1DM patients, respectively, according to a meta-analysis study^4^. Clinically, the relationship between DPN and RAS is not necessarily clear, and the clinical efficacy of RAS inhibitors in reducing DPN is still open to question.

Ang I is formed from angiotensinogen by renin, and converted into Ang II by angiotensin-converting enzyme (ACE), which activates AT_1_ and AT_2_ receptors. The peripheral RAS plays an important role in the regulation of blood pressure and fluid volume, and AT_1_ receptor blockers (angiotensin receptor blockers; ARBs) and ACE inhibitors (ACEIs) are clinically used to treat or prevent hypertension, diabetic nephropathy and chronic heart failure. Most interestingly, accumulating evidence suggests that an Ang system like peripheral RAS is present in the brain and spinal cord, where angiotensinogen, Ang I, Ang II, ACE, AT_1_ and AT_2_ receptors, and cathepsin D, a renin-like enzyme, are detectable^5^. In the CNS, supraspinal Ang II is considered to promote nociception via activation of AT_1_ and/or AT_2_ receptors, although AT_1_ receptor activation in a certain brain area appears to suppress nociception^5^. The functional upregulation of the spinal Ang II/AT_1_ receptor system is involved in T1DM-related DPN^6^. In contrast, the MAS receptors activated by Ang (1–7) formed from Ang II by ACE2 might function to rather reduce DPN in a T2DM model^7^. In the primary afferents including the dorsal root ganglion (DRG) and sciatic nerves, the Ang II/AT_1_ receptor system may contribute to the development of neuropathic pain^5^. It is to be noted that clinical and preclinical studies have suggested the involvement of the Ang II/AT_1_ receptor system in the chemotherapy-induced peripheral neuropathy^8–10^. Activation of AT_2_ receptors expressed in macrophages is also implicated in neuropathic pain^11,12^, and the effectiveness of AT_2_ receptor antagonists against neuropathic pain has been demonstrated in clinical and preclinical studies^13–15^. Collectively, the peripheral and/or central Ang II is considered to play a role in the development of neuropathic pain including DPN. It is thus likely that, of DM patients undergoing antihypertensive pharmacotherapy, ones receiving RAS blockers such as ACEIs and ARBs might have a lower risk than ones receiving the other (non-ACEI, non-ARB) antihypertensive agents. To test this hypothesis, in the present study, we conducted a retrospective cohort study by collecting medical information of T2DM patients undergoing antihypertensive medications at three different hospitals in Japan over a period of approximately a decade. On the basis of the results from the clinical study, we also performed a reverse translational study to ask whether ACEIs and ARBs could prevent the development of DPN in leptin-deficient *ob*/*ob* mice, a model of T2DM.

## Methods

### Laboratory animals employed and ethical approval of the experimental procedures in a reverse translational study

Leptin-deficient *ob*/*ob* mice exhibit obesity and the resulting metabolic abnormalities, such as insulin resistance, hyperinsulinemia, and hyperglycemia, with a phenotype similar to human T2DM^16,17^. Male *ob*/*ob* mice, the T2DM model, and age-matched lean (*ob*/+ or +/+) mice are obtained from CLEA Japan, Inc (Tokyo, Japan), and housed in standard shoebox cages located in a room maintained at a temperature of 22 ± 2 °C under a 12/12-h light/dark cycle (lights on: 07:00), with free access to food and tap water. All procedures for animal experiments conformed to National Institutes of Health Guide for the Care and Use of Laboratory Animals, and were approved by Ethics Committee of Animal Experiment at Tohoku Medical and Pharmaceutical University. The present study is reported in accordance with the ARRIVE guidelines (https://arriveguidelines.org).

### Protocol of animal experiments

Nociceptive sensitivity in *ob*/*ob* and the control lean mice was determined from the age of 5 until 12 weeks old. Mechanical nociceptive sensitivity was evaluated by the up-down method^18^ using a set of 8 calibrated von Frey filaments (Stoelting Touch Test Sensory Evaluator Kit #2 to #9: ranging from ≈ 0.018 to ≈ 1.4 g of force). Mice were placed within Plexiglas cubicles (9 × 5 × 5 cm high) that were positioned atop a perforated metal floor. The von Frey filaments were applied perpendicularly against the plantar surface of hindpaw until the fibers bowed, and then held for 3 s. A withdrawal from the filament, or lack thereof, was observed within the 3-s time window. At every time point, withdrawal threshold was measured once in each of the left and right hind paws, and the obtained two values were averaged. Thermal nociceptive sensitivity was measured using a plantar analgesia meter (Model 390; IITC Life Sciences, Los Angeles, CA, USA). Mice were placed in the above mentioned cubicles (9 × 5 × 5 cm high) positioned atop a 0.5-cm-think glass plate and habituated for at least 1 h before testing. A mobile high-intensity halogen lamp beam placed under the glass floor was focused on the plantar hind paw. The device was set to 20% of the maximum available heat intensity of the device (≈ 35 W/mm^2^). Withdrawal latencies were measured three times in each of the left and right hind paws, and the obtained six values were averaged. The assay of mechanical and thermal nociceptive sensitivity was carried out before drug administration on each day.

The minimum necessary amount of blood was withdrawn from the tail vein with a 27G needle after disinfection of the mouse tail with 80% ethanol every week, and glucose levels were measured with a FreeStyle Precision Neo-Blood Glucose Monitoring Meter (Abbott Japan, Tokyo, Japan).

Three different antihypertensive agents, i.e. perindopril erbumine (Sigma-Aldrich, St. Louis, MO, USA), an ACEI, telmisartan (FUJIFILM Wako Pure Chemical, Osaka, Japan), an ARB, and amlodipine besylate (Tokyo Chemical Industry, Tokyo, Japan), an L-type calcium channel blocker (CaB), were used to test their effects on the development of DPN in the *ob*/*ob* mice. The appropriate doses of these drugs in mice were decided according to the previous reports^19–21^. Perindopril and amlodipine were dissolved in saline, and telmisartan was in saline containing 5% DMSO, 5% Tween-80 and 20% polyethylene glycol 300. Perindopril at 2 mg/kg, telmisartan at 5 mg/kg and amlodipine at 3 mg/kg, in a volume of 0.1 mg/10 g body weight, were administered i.p. to *ob*/*ob* mice once a day for 6 weeks (i.e. until the age of 12 weeks), starting at the age of 6 weeks. All mice used in the present study were euthanized by CO_2_ inhalation after the experiments.

### Data analysis for animal experiments

The data obtained from animal experiments are shown as means ± SEM. Statistical significance was evaluated by analysis of variance followed by Tukey’s test for multiple comparisons of parametric data, and by Kruskal–Wallis *H*-test followed by a least significant difference-type test for multiple comparisons of non-parametric data.

### T2DM patients enrolled and the inclusion/exclusion criteria in a clinical retrospective cohort study

We collected medical record information of 9478 ambulatory patients with T2DM who received antihypertensive medications at Kansai Medical University Hospital, Kindai University Nara Hospital and Seichokai Fuchu Hospital from April 2011 to December 2020. The observation period was from the first medication for T2DM (i.e. sulfonylureas, glinides, biguanides, α-glucosidase inhibitors, sodium-glucose transporter 2 inhibitors, dipeptidyl peptidase 4 inhibitors, glucagon-like peptides 1 receptor agonists, thiazolidinediones) at each hospital until the diagnosis of DPN or the last hospital visit. Exclusion criteria (Supplementary Fig. 1) were as follows: (1) DM medications for less than 7 days (n = 1647), (2) diagnosis of neuropathy prior to DM diagnosis (n = 327), and (3) under 20 years of age (n = 40). DPN in T2DM patients was diagnosed by a physician at intervals of 2–12 weeks, according to the diagnostic criteria of diabetic neuropathies^22^ and patient’s complaints. Thus, 7464 patients who met inclusion criteria were enrolled in this study, and the collected information from them included antihypertensive medications listed in the latest prescriptions, i.e. ARBs, ACEIs, CaBs, β-blockers or thiazide/thiazide-like diuretics, and the latest laboratory test values including hemoglobin A1c-national glycohemoglobin standardization program (HbA1c-NGSP), serum creatinine (Scr) and C-reactive protein (CRP) during the observation period.

### Clinical research design and statistical analysis in the retrospective cohort study

The enrolled T2DM patients with hypertension were divided into three groups according to the prescribed antihypertensive medications: (1) ACEIs, (2) ARBs and (3) others, i.e. non-ACEI, non-ARB antihypertensive agents including CaBs, β-blockers and thiazide/thiazide-like diuretics. The time-related incidence of DPN in the three groups during the observation period was examined, compared by generating Kaplan–Meier curves and analyzed by Log-rank test. Bonferroni’s test was used for multiple comparisons between the three groups. Sub-analyses of the data obtained at each of the three hospitals and of the data from the two patient groups receiving blood–brain-barrier (BBB)-permeable and BBB-impermeable ACEI or ARB, respectively, were also performed. It is to be noted that the patients receiving both ACEI and ARB (n = 25) were not included in this analysis.

Univariate and multivariate analyses using a Cox proportional hazard model were conducted to statistically evaluate the association of variables including the initial age, the latest values of CRP, HbA1c-NGSP, Scr (the median and over) during the observation period, gender (female), and the prescription of ACEIs or ARBs, CaBs, β-blockers and thiazide/thiazide-like diuretics with the time-related DPN development. The association of patient information including smoking history, serum lipid profile and blood pressure values, which could be obtained only from Kansai Medical University and Kindai University hospitals, with DPN development was evaluated by sub-analysis of the two hospitals’ medical data. Collinearity was examined with a variance inflation factor (VIF). The variable we used for all multivariate analyses was VIF < 5. HR for each variable is shown with 95% CI.

A *p* value less than 0.05 was considered statistically significant in clinical analysis. EZR^23^, a pilot user interface for R (version 1.61), was employed for statistical analysis of clinical data.

### Ethical approval of the clinical study

The clinical study protocol was in accordance with the relevant guidelines and regulations including the Declaration of Helsinki, and approved by Ethics Committees of Kansai Medical University (approval number 2021347), Kindai University Nara Hospital (approval number 667) and Seichokai Fuchu Hospital (approval number 2022002). Considering the retrospective nature of this study, the need for informed consent was waived by each of the above-described three Ethics Committees, which approved the use of an opt-out procedure concerning patient consent, i.e. study patients were enrolled in the clinical analysis unless the individual patient requested an exemption. The official website of each hospital was used for communication of the research information with the patients. Any of our research members named in the author list did not have access to the information necessary for identifying individuals when analyzing the data.

## Results

### Reverse translational analysis of the effect of the Ang system inhibition on DPN development in leptin-deficient *ob*/*ob* mice, a model for T2DM

**Figure 1:**
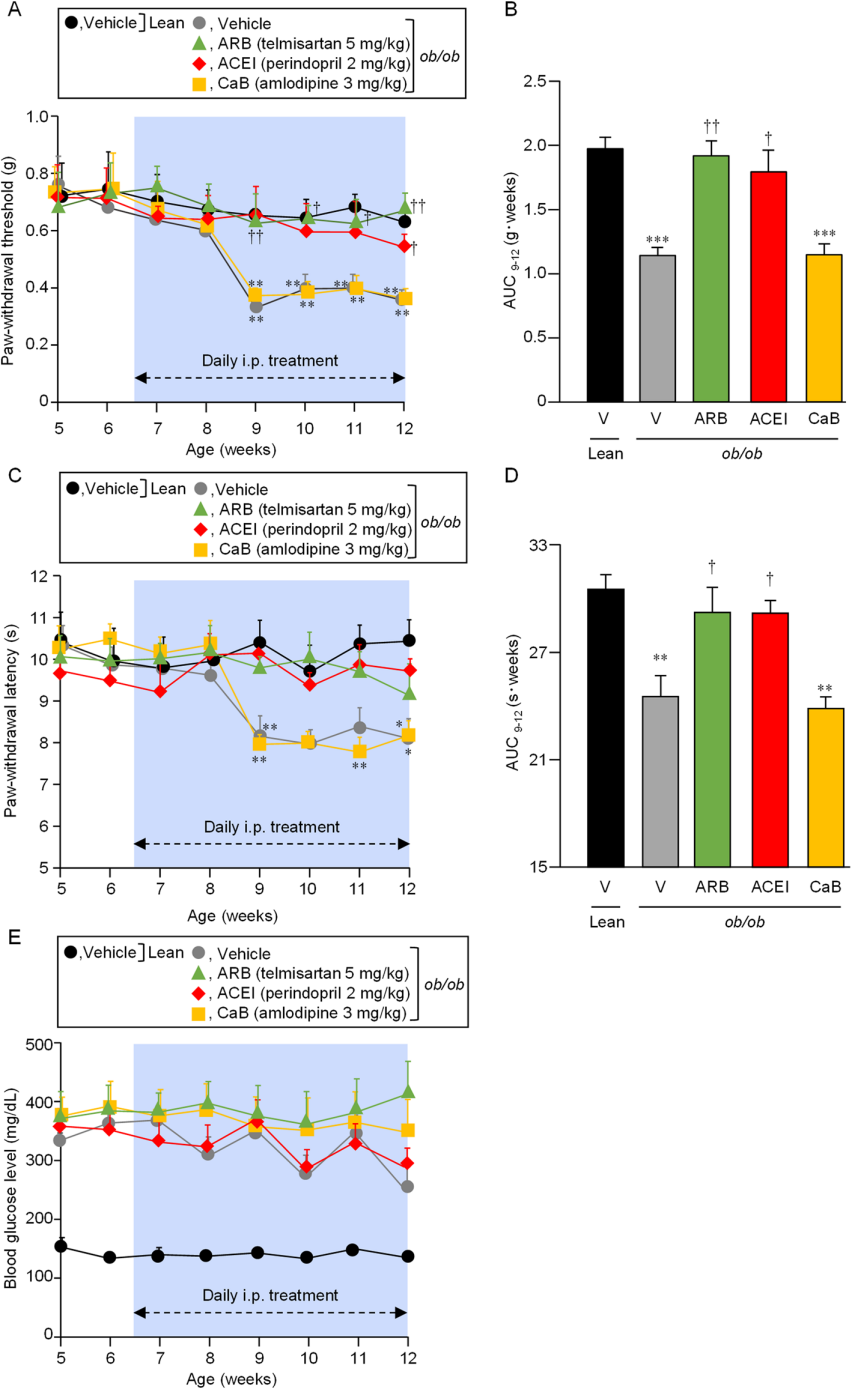
Effects of daily treatment with representative ARB, ACEI and CaB on the development of DPN in leptin-deficient *ob*/*ob* mice. Tactile (**A** and **B**) and thermal (**C** and **D**) nociceptive sensitivities and blood glucose levels (**E**) were determined in *ob*/*ob* mice and the control age-matched lean mice between 5 and 12 weeks of age. Telmisartan at 5 mg/kg, perindopril at 2 mg/kg or amlodipine at 3 mg/kg was administered i.p. to *ob*/*ob* mice once daily for 6 weeks (i.e. until the age of 12 weeks), starting at the age of 6 weeks. AUC values between 9 and 12 weeks of age, (**B**) and (**D**), were calculated from the time-related changes in paw-withdrawal threshold (**A**) and paw-withdrawal latency (**C**). Values represent the means ± SEM for 8 mice per group.

On the basis of the clinical evidence for the negative association of the prescription of ACEIs and ARBs, but not CaBs, with DPN development in the above-described retrospective cohort study, we conducted a reverse translational analysis using leptin-deficient *ob*/*ob* mice, a model for T2DM. Perindopril, an ACEI, at 2 mg/kg, telmisartan, an ARB, at 5 mg/kg, and amlodipine, a CaB, at 3 mg/kg, were administered i.p. daily to *ob*/*ob* mice for 6 weeks, starting at 6 weeks of age. The *ob*/*ob* mice at the age of 6 weeks already had maximal hyperglycemia, i.e. blood glucose levels higher than 300 mg/dL (Fig. 1E), but not DPN, compared to the control lean mice (Fig. 1A, C). Thereafter, they clearly developed DPN, i.e. decreased paw-withdrawal threshold (g) and shortened latency (s) in response to mechanical and thermal nociceptive stimuli, respectively, from the age of 9 weeks at least until 12 weeks (Fig. 1A, C). Interestingly, the *ob*/*ob* mice subjected to daily i.p. treatment with the ARB or ACEI developed neither mechanical nor thermal nociceptive hypersensitivity between the age of 9 and 12 months (Fig. 1A–D), although their blood glucose levels remained maximally elevated during and after treatment with ARB or ACEI (Fig. 1E). In contrast, the *ob*/*ob* mice receiving daily CaB administration had DPN development at the age of 9–12 weeks, in addition to the antecedent hyperglycemia, as the vehicle-treated *ob*/*ob* mice did (Fig. 1).

### Patient characteristics in a clinical retrospective cohort study

Of 9478 patients with T2DM undergoing antihypertensive pharmacotherapy at Kansai Medical University Hospital, Kindai University Nara Hospital and Seichokai Fuchu Hospital in Japan for nearly a decade, from April 2011 to December 2020, 7464 in consideration of inclusion/exclusion criteria were enrolled in this study (Table 1). The overall DPN incidence was 12% after the observation periods, and the median (range) of the initial age (years) and the latest values of HbA1c-NGSP (%), Scr and CRP (mg/dL) during the observation periods were 73 (23–106), 6.8 (2.9–17), 0.9 (0.17–18) and 0.23 (0–39), respectively (Table 1). It is to be noted that the median of baseline HbA1c-NGSP was 7.2%, and that the median of observation periods was 373 days. The major categories of antihypertensive agents in the order of prescription frequency were CaBs > ARBs > β-blockers > ACEIs > thiazide/thiazide-like diuretics (Table 1).

**Table 1:** Characteristics of the enrolled T2DM patients undergoing antihypertensive pharmacotherapy who met inclusion criteria.

Characteristics	Numerical data
Patients who met inclusion criteria	
Total, n	7464
Kansai Med Univ Hosp, n (%)	3945 (53)
Kindai Univ Nara Hosp, n (%)	2047 (27)
Seichokai Fuchu Hosp, n (%)	1472 (20)
Initial age (years) during the observation period, median (range)	73 (23–106)
Gender, female/male, n (%)	2509 (34)/4955 (66)
DPN development during the observation period, n (%)	868 (12)
Latest laboratory data during the observation period	
HbA1c-NGSP (%), median (range), n	6.8 (2.9–17), 6383
Scr (mg/dL), median (range), n	0.9 (0.17–18), 7194
CRP (mg/dL), median (range), n	0.23 (0–39), 6438
Prescribed antihypertensive agents	
ARB, n (%)	3983 (53)
ACEI, n (%)	976 (13)
CaB, n (%)	5213 (70)
β-blocker, n (%)	1833 (25)
Thiazide/thiazide-like diuretics, n (%)	703 (10)

The data were collected from medical records of the patients at Kansai Medical (Med) University (Univ) Hospital (Hosp), Kindai Univ Nara Hosp or Seichokai Fuchu Hosp. The median of the observation period (from the first medication for T2DM until the diagnosis of DPN or the last hospital visit) was 373 days, and the median of the initial HbA1c-NGSP was 7.2% (range: 3.3–18.3). DPN, diabetic peripheral neuropathy; HbA1c-NGSP, hemoglobin A1c-national glycohemoglobin standardization program; Scr, serum creatinine; CRP, C-reactive protein; ARB, angiotensin receptor blocker; ACEI, angiotensin-converting enzyme inhibitor; CaB, L-type calcium channel blocker.

### Association of the prescription of ACEIs or ARBs with the development of DPN in T2DM patients undergoing antihypertensive medications at the three hospitals

**Figure 2:**
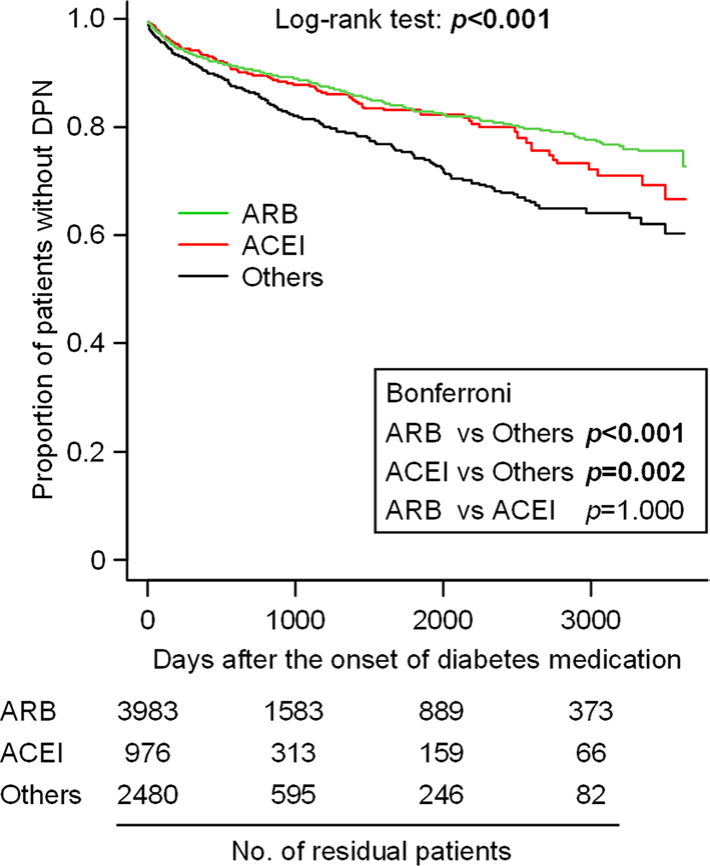
Kaplan–Meier curves for the development of DPN in T2DM patients undergoing different antihypertensive pharmacotherapies at the three hospitals. The patients were divided into 3 groups according to the prescribed antihypertensives, i.e. (1) ACEI, (2) ARB and (3) Others (non-ACEI, non-ARB antihypertensives). In this analysis, 25 patients receiving both ACEI and ARB were not included. Statistical significance was analyzed by Log-rank test, followed by Bonferroni’s test for multiple comparisons.

**Figure 3:**
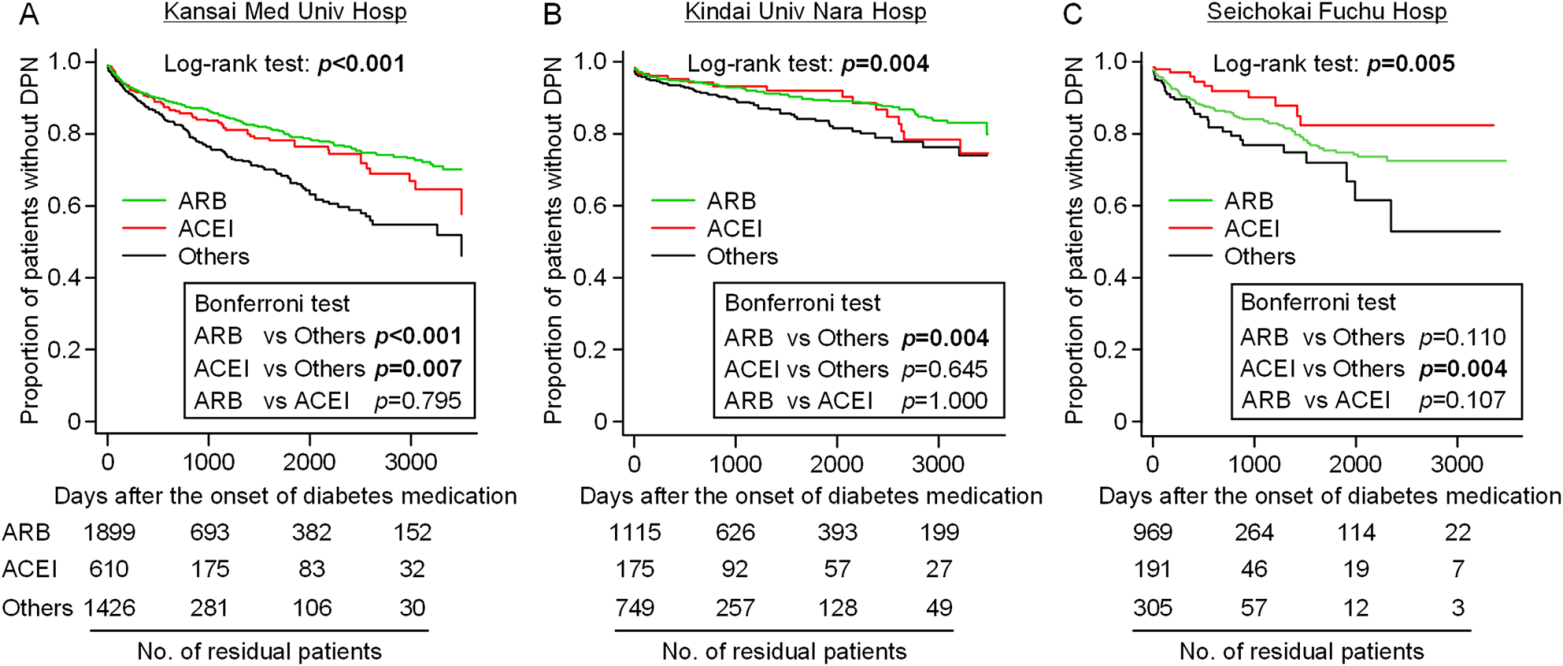
Sub-analysis of the association of the prescribed antihypertensives with the development of DPN in T2DM patients at each of the three hospitals. Kaplan–Meier curves of the three patient groups receiving ACEI, ARB and the others (non-ACEI, non ARB antihypertensives) were drawn for each of Kansai Medical (Med) University (Univ) Hospital (Hosp) (**A**), Kindai Univ Nara Hosp (**B**) or Seichokai Fuchu Hosp (**C**). Statistical significance was analyzed by Log-rank test, followed by Bonferroni’s test for multiple comparisons.

Study patients were divided into three groups according to the pharmacological classes of the prescribed antihypertensive agents, i.e. ARB (ARB alone or in combination with non-ACEI antihypertensive agents), ACEI (ACEI alone or in combination with non-ARB antihypertensive agents) and “others” (non-ARB and non-ACEI antihypertensive agents), and the differences of the time-related incidence of DPN between the 3 groups were statistically analyzed. Kaplan–Meier curves and the log-rank test showed clearly and significantly (*p* < 0.001) different development of DPN among the 3 groups (Fig. 2). Bonferroni’s multiple comparison test indicated significantly delayed development of DPN in the patient groups treated with ARBs or ACEIs, compared to “others (non-ARB and non-ACEI antihypertensives)”, and no significant difference of DPN development between ARB and ACEI groups (Fig. 2). Sub-analysis of the data of each hospital showed a similar tendency, i.e. delayed development of DPN in ARB and ACEI groups in comparison with “others” (Fig. 3). These results indicate that the incidence of DPN in T2DM patients may be suppressed or delayed by ACEIs or ARBs.

To ask whether the central or peripheral Ang II/AT_1_ receptor system is involved in the DPN development, a sub-analysis of the data of the T2DM patients receiving ARBs or ACEIs was conducted in terms of their BBB permeability. ACEs and ARBs were divided into 2 classes according to their BBB permeability, i.e. BBB-permeable ARB/ACEI (candesartan, valsartan, telmisartan, azilsartan, captopril, perindopril, lisinopril, temocapril) and BBB-impermeable ARB/ACEI (losartan, irbesartan, olmesartan, enalapril, quinapril, imidapril)^24–30^. Kaplan–Meier curves and the log-rank test showed no difference of DPN development between the two DM groups receiving BBB-permeable and BBB-impermeable ARB/ACEI (Supplementary Fig. 2).

We then used univariate and multivariate Cox proportional hazard regression models to evaluate the effect of diverse factors including the use of ARB or ACEI on the development of DPN in patients with T2DM. The univariate analysis detected significant positive or negative association of ages ≥ 73 years (median) [hazard ratio (HR), 1.48; 95% confidence interval (CI), 1.30–1.70; *p* < 0.001], female (HR, 1.41; 95% CI, 1.23–1.62; *p* < 0.001), CRP ≥ 0.23 mg/dL (median) (HR, 1.30; 95% CI; 1.13–1.49; *p* < 0.001), Scr ≥ 0.9 mg/dL (median) (HR, 0.95; 95% CI 0.91–1.00; *p* = 0.042), and the prescription of ACEI or ARB (HR, 0.63; 95% CI 0.55–0.73; *p* < 0.001), β-blocker (HR, 0.66; 95% CI 0.56–0.78; *p* < 0.001) and thiazide/thiazide-like diuretics (HR, 0.75; 95% CI 0.59–0.94; *p* = 0.014) with the development of DPN (Table 2). Then, the multivariate analysis indicated independently significant positive association of age ≥ 73 years (HR, 1.38; 95% CI 1.18–1.61; *p* < 0.001) and CRP ≥ 0.23 mg/dL (HR, 1.28; 95% CI 1.10–1.49; *p* = 0.002), and independently significant negative association of the prescription of ACEIs or ARBs (HR, 0.64; 95% CI 0.55–0.76; *p* < 0.001) and β-blocker (HR, 0.69; 95% CI 0.57–0.82;* p* < 0.001) with DPN development (Table 2). In contrast, there was no impact of the prescription of CaBs or thiazide/thiazide-like diuretics on DPN development (Table 2). Considering the β-blocker-induced suppression of sympathetically mediated renin release, the inhibition of the renin-angiotensin system has something to do with the prevention or delay of DPN development in T2DM patients.

**Table 2:** Cox proportional univariate and multivariate analyses of the association with variables including the latest laboratory test results and prescribed antihypertensive agents with DPN development.

Variables	Univariate analysis		Multivariate analysis	
	Hazard ratio (95% CI)	*p* value	Hazard ratio (95% CI)	*p* value
Age, ≥ 73 years (median)	1.48 (1.30–1.70)	** < 0.001**	1.38 (1.18–1.61)	** < 0.001**
Gender, female	1.41 (1.23–1.62)	** < 0.001**	0.98 (0.83–1.16)	0.83
HbA1c-NGSP, ≥ 6.8% (median)	0.96 (0.83–1.11)	0.6	0.88 (0.76–1.03)	0.12
Scr, ≥ 0.9 mg/dL (median)	0.95 (0.91–1.00)	**0.042**	0.89 (0.75–1.04)	0.14
CRP, ≥ 0.23 mg/dL (median)	1.30 (1.13–1.49)	** < 0.001**	1.28 (1.10–1.49)	**0.002**
ARB or ACEI	0.63 (0.55–0.73)	** < 0.001**	0.64 (0.55–0.76)	** < 0.001**
CaB	1.08 (0.93–1.25)	0.33	0.87 (0.73–1.03)	0.12
β-blocker	0.66 (0.56–0.78)	** < 0.001**	0.69 (0.57–0.82)	** < 0.001**
Thiazide/thiazide-like diuretics	0.75 (0.59–0.94)	**0.014**	0.82 (0.63–1.07)	0.14

CI, confidence interval. HbA1c-NGSP, hemoglobin A1c-national glycohemoglobin standardization program; Scr, serum creatinine; CRP, C-reactive protein; ARB, angiotensin receptor blocker; ACEI, angiotensin-converting enzyme inhibitor; CaB, L-type calcium channel blocker.

Significant values are in bold.

Patient information such as smoking history, serum lipids and blood pressure was available from the medical data of Kansai Medical University Hospital and Kindai University Hospital, but not Seichokai Fuchu Hospital. We thus conducted a sub-analysis of the data in the two hospitals, using patient information including the history of smoking and the latest values of serum lipids and blood pressure during the observation periods. The univariate Cox proportional analysis detected the use of ARB or ACEI as a factor that significantly reduced the risk of DPN, and showed that low-HDL cholesterol and diastolic blood pressure values were slightly, but significantly, associated with DPN development (Supplementary Table 1). On the other hand, multivariate Cox proportional analysis demonstrated that the use of ARB or ACEI, but not the other variables, was significantly associated with the decreased development of DPN, which was independent of the history of smoking, abnormal serum lipid profile or blood pressure values (Supplementary Table 1).

## Discussion

Our data from the clinical retrospective cohort study in T2DM patients undergoing antihypertensive pharmacotherapy at 3 hospitals in Japan demonstrated significant negative association of the prescription of ACEIs or ARBs as well as β-blockers with the development of DPN. This clinical finding was supported by our preclinical study, in which daily treatment with ACEI or ARB, but not CaB, prevented the development of DPN without affecting the hyperglycemia in leptin-deficient *ob*/*ob* mice, a T2DM model. Thus, our study suggests that pharmacological inhibition of the β-adrenoceptor/renin/Ang pathway is beneficial to prevent the development of DPN, in addition to diabetic nephropathy and cardiomyopathy^3,31,32^, in T2DM patients.

A randomized double-blind controlled trial showed that treatment with trandolapril, an ACEI, for 6–12 months tended to improve peripheral neuropathy in 41 normotensive patients with T1DM or T2DM^33^. However, no further information about clinical effectiveness of ACEIs or ARBs on DPN development is available. Several studies reported that hypertension itself might increase the risk of neuropathy^34–36^, whereas, in randomized controlled trials in DM patients, more aggressive anti-hypertensive treatment did not prevent or delay the progression of neuropathy, compared with less strict blood pressure controls^37,38^. In the present study, T2DM patients undergoing antihypertensive pharmacotherapy were enrolled in the retrospective analysis, in order to minimize the effect of hypertension itself on DPN development. The findings of particular interest are that T2DM patients receiving antihypertensive medications other than ACEIs and ARBs had significantly earlier development of DPN than ones receiving ACEIs or ARBs, as analyzed by Bonferroni’s test (see Figs. 2 and 3), and that, unlike ACEIs/ARBs or β-blockers, the prescription of CaBs or thiazide/thiazide-like diuretics did not have significant impact on DPN development in T2DM patients, as evaluated by Cox proportional multivariate analysis (see Table 2). It is also noteworthy that the multivariate sub-analysis of the patient information including blood pressure in the two hospitals clearly indicated that the preventive effects of ACEI or ARB on DPN development is independent of systolic and diastolic blood pressure values in addition to the history of smoking and abnormal serum lipid profile (see Supplementary Table 1). These clinical results are essentially in agreement with the findings from the animal experiments that daily treatment with the ACEI or ARB, but not CaB, prevented the development of DPN in the *ob*/*ob* mice, a model for T2DM (see Fig. 1).

The D allele of the ACE insertion/deletion (I/D) gene variant, which is associated with higher ACE activity, could be related to increased risk of DPN as well as diabetic nephropathy in Caucasian^39,40^. Angiotensinogen (AGT) M235T variant may be associated with myocardial infarction and brain infarction in East Asian group^41^. Most interestingly, a study in the Japanese population has provided controversial evidence that the D allele of the ACE I/D has a protective effect on polyneuropathy, while there is no association between AGT gene polymorphism and polyneuropathy^42^. This discrepancy is still open to question.

The molecular mechanisms underlying the ACEI/ARB-induced suppression of DPN development accompanying T2DM in the clinical and preclinical studies are still open to question. Accumulating evidence suggests involvement of both central and peripheral Ang systems in pain processing^5,43^. However, the Ang system in the CNS, if any, is considered to play a relatively minor role in DPN development accompanying T2DM in the present study, because there was no difference of DPN development between two T2DM patient groups receiving BBB-permeable and BBB-impermeable ACEIs/ARBs, respectively, in the clinical study (see supplementary Fig. 2). There is evidence that Ang II-induced activation of both peripheral AT_1_ and AT_2_ receptors participates in the development of neuropathic pain^5,9–14^. In the present clinical study, however, the prescription of ARBs, i.e. AT_1_ receptor antagonists, had significant impact on DPN development that was equal to or greater than ACEIs in T2DM patients (see Figs. 2 and 3), suggesting the essential role of the Ang II/AT_1_ receptor system in DPN development. The downstream signals of AT_1_ receptor activation involved in DPN development remain to be investigated in future studies, although stimulation of AT_1_ receptors is known to cause oxidative stress^44^ which could be involved in the pathogenesis of DPN^45^. In our clinical study, the number of T2DM patients receiving aliskiren, a renin inhibitor, was quite limited, so that the effect of aliskiren on DPN development could not be analyzed. Nonetheless, it is to be noted that an animal study demonstrated the beneficial effect of aliskiren in attenuating DPN in streptozotocin-induced diabetic rats^46^. Considering the significant impact of β-blockers on DPN development in the clinical study (see Table 2), the peripheral β-adrenoceptor/renin/Ang/AT_1_ receptor system appears to participate in DPN development accompanying T2DM, and may serve as therapeutic targets for DPN.

An animal model of T1DM is created by single or a few administrations of streptozocin (STZ) in mice, and widely used in fundamental studies on complications of T1DM. However, it has been reported that STZ activates transient receptor potential (TRP) channels at the peripheral nerve level in a hyperglycemia-independent manner, implying that this DM model might not be suitable for studying DPN^47,48^. Also, in the clinical study, we did not determine the effects of ACEIs or ARBs on DPN accompanying T1DM, because of the insufficient number of T1DM patients.

This work had some strengths and limitations. Information from medical records of 7464 T2DM patients undergoing antihypertensive pharmacotherapy at 3 hospitals was subjected to statistical analysis, and the obtained clinical evidence was then confirmed by a reverse translational study using an animal model for T2DM. The limitation of our clinical study includes the retrospective nature and the lack of detailed analysis of the effects of time-related blood pressure control and dosage of antihypertensive agents on DPN development. In future, prospective studies and/or meta-analysis should be conducted to ascertain the present findings.

In conclusion, the present combined clinical and reverse translational approaches unveiled the essential role of the peripheral β-adrenoceptor/renin/Ang/AT_1_ receptor system in the development of DPN accompanying T2DM, suggesting the therapeutic usefulness of its inhibition for prevention of DPN development. The prescription of ACEIs, ARBs or β-blockers, rather than CaBs or diuretics, is thus recommended to prevent DPN in T2DM patients.

### Supplementary Information

Supplementary Information.

## Data Availability

The data used in the present study are available form the corresponding author on reasonable request.

## References

[1] Shi M (2023). Identification of candidate metabolite biomarkers for metabolic syndrome and its five components in population-based human cohorts. Cardiovasc. Diabetol..

[2] Wang Z (2017). Synergistic interaction of hypertension and diabetes in promoting kidney injury and the role of endoplasmic reticulum stress. Hypertension.

[3] Banerjee D (2022). Management of hypertension and renin-angiotensin-aldosterone system blockade in adults with diabetic kidney disease: Association of British clinical diabetologists and the renal association UK guideline update 2021. BMC Nephrol..

[4] Sun J, Wang Y, Zhang X, Zhu S, He H (2020). Prevalence of peripheral neuropathy in patients with diabetes: A systematic review and meta-analysis. Prim. Care Diabetes.

[5] Nemoto W, Yamagata R, Nakagawasai O, Tan-No K (2023). Angiotensin-related peptides and their role in pain regulation. Biology.

[6] Ogata Y (2016). Involvement of spinal angiotensin II system in streptozotocin-induced diabetic neuropathic pain in mice. Mol. Pharmacol..

[7] Yamagata R, Nemoto W, Nakagawasai O, Takahashi K, Tan-No K (2020). Downregulation of spinal angiotensin converting enzyme 2 is involved in neuropathic pain associated with type 2 diabetes mellitus in mice. Biochem. Pharmacol..

[8] Frachet S (2022). Renin-angiotensin-system inhibitors for the prevention of chemotherapy-induced peripheral neuropathy: OncoToxSRA, a preliminary cohort study. J. Clin. Med..

[9] Kim E, Hwang SH, Kim HK, Abdi S, Kim HK (2019). Losartan, an angiotensin II type 1 receptor antagonist, alleviates mechanical hyperalgesia in a rat model of chemotherapy-induced neuropathic pain by inhibiting inflammatory cytokines in the dorsal root ganglia. Mol. Neurobiol..

[10] Kalynovska N, Diallo M, Sotakova-Kasparova D, Palecek J (2020). Losartan attenuates neuroinflammation and neuropathic pain in paclitaxel-induced peripheral neuropathy. J. Cell Mol. Med..

[11] Shepherd AJ (2018). Angiotensin II triggers peripheral macrophage-to-sensory neuron redox crosstalk to elicit pain. J. Neurosci..

[12] Shepherd AJ (2018). Macrophage angiotensin II type 2 receptor triggers neuropathic pain. Proc. Natl. Acad. Sci..

[13] Rice ASC (2014). EMA401, an orally administered highly selective angiotensin II type 2 receptor antagonist, as a novel treatment for postherpetic neuralgia: A randomised, double-blind, placebo-controlled phase 2 clinical trial. Lancet.

[14] Rice ASC (2021). Efficacy and safety of EMA401 in peripheral neuropathic pain: Results of 2 randomised, double-blind, phase 2 studies in patients with postherpetic neuralgia and painful diabetic neuropathy. Pain.

[15] Khan N, Muralidharan A, Smith MT (2017). Attenuation of the infiltration of angiotensin II expressing CD3^+^ T-cells and the modulation of nerve growth factor in lumbar dorsal root ganglia: A possible mechanism underpinning analgesia produced by EMA300, an angiotensin II type 2 (AT_2_) receptor antagonist. Front. Mol. Neurosci..

[16] Genuth SM, Przybylski RJ, Rosenberg DM (1971). Insulin resistance in genetically obese, hyperglycemic mice. Endocrinology.

[17] Zhang Y (1994). Positional cloning of the mouse obese gene and its human homologue. Nature.

[18] Chaplan SR, Bach FW, Pogrel JW, Chung JM, Yaksh TL (1994). Quantitative assessment of tactile allodynia in the rat paw. J. Neurosci. Methods.

[19] Tikellis C (2008). ACE2 deficiency modifies renoprotection afforded by ACE inhibition in experimental diabetes. Diabetes.

[20] Fujita H (2012). Modulation of renal superoxide dismutase by telmisartan therapy in C57BL/6-Ins2(Akita) diabetic mice. Hypertens. Res..

[21] Yoshii T (2006). Regression of atherosclerosis by amlodipine via anti-inflammatory and anti-oxidative stress actions. Hypertens. Res..

[22] Tesfaye S (2010). Diabetic neuropathies: Update on definitions, diagnostic criteria, estimation of severity, and treatments. Diabetes Care.

[23] Kanda Y (2013). Investigation of the freely available easy-to-use software 'EZR' for medical statistics. Bone Marrow Transplant..

[24] Gao Q (2017). Azilsartan ameliorates apoptosis of dopaminergic neurons and rescues characteristic parkinsonian behaviors in a rat model of Parkinson's disease. Oncotarget.

[25] Noda A (2012). Brain penetration of telmisartan, a unique centrally acting angiotensin II type 1 receptor blocker, studied by PET in conscious rhesus macaques. Nucl. Med. Biol..

[26] Dong YF (2011). Perindopril, a centrally active angiotensin-converting enzyme inhibitor, prevents cognitive impairment in mouse models of Alzheimer's disease. FASEB J..

[27] Nozawa M, Sugimoto K, Ohmori M, Ando H, Fujimura A (2006). Dosing time-dependent effect of temocapril on the mortality of stroke-prone spontaneously hypertensive rats. J. Pharmacol. Exp. Ther..

[28] Takai S, Jin D, Sakaguchi M, Miyazaki M (2004). Significant target organs for hypertension and cardiac hypertrophy by angiotensin-converting enzyme inhibitors. Hypertens. Res..

[29] Dobrowolski C (2023). Centrally acting ACE inhibitor (cACEi) and angiotensin receptor blocker (cARB) use and cognitive dysfunction in patients with SLE. Lupus Sci. Med..

[30] Sink KM (2009). Angiotensin-converting enzyme inhibitors and cognitive decline in older adults with hypertension: Results from the cardiovascular health study. Arch. Intern. Med..

[31] Sugahara M, Pak WLW, Tanaka T, Tang SCW, Nangaku M (2021). Update on diagnosis, pathophysiology, and management of diabetic kidney disease. Nephrology.

[32] Sukumaran V, Gurusamy N, Yalcin HC, Venkatesh S (2022). Understanding diabetes-induced cardiomyopathy from the perspective of renin angiotensin aldosterone system. Pflugers Arch..

[33] Malik RA (1998). Effect of angiotensin-converting-enzyme (ACE) inhibitor trandolapril on human diabetic neuropathy: Randomised double-blind controlled trial. Lancet.

[34] Tesfaye S (2005). Vascular risk factors and diabetic neuropathy. N. Engl. J. Med..

[35] Forrest KY, Maser RE, Pambianco G, Becker DJ, Orchard TJ (1997). Hypertension as a risk factor for diabetic neuropathy: A prospective study. Diabetes.

[36] Ponirakis G (2019). Hypertension contributes to neuropathy in patients with type 1 diabetes. Am. J. Hypertens..

[37] Schrier RW, Estacio RO, Esler A, Mehler P (2002). Effects of aggressive blood pressure control in normotensive type 2 diabetic patients on albuminuria, retinopathy and strokes. Kidney Int..

[38] Patel A (2007). Effects of a fixed combination of perindopril and indapamide on macrovascular and microvascular outcomes in patients with type 2 diabetes mellitus (the ADVANCE trial): A randomised controlled trial. Lancet.

[39] Stephens JW, Dhamrait SS, Acharya J, Humphries SE, Hurel SJ (2006). A common variant in the ACE gene is associated with peripheral neuropathy in women with type 2 diabetes mellitus. J. Diabetes Complicat..

[40] Rahimi Z (2012). ACE insertion/deletion (I/D) polymorphism and diabetic nephropathy. J. Nephropathol..

[41] Liang X (2013). Polymorphism of angiotensinogen gene M235T in myocardial infarction and brain infarction: A meta-analysis. Gene.

[42] Ito H (2002). Angiotensin-converting enzyme insertion/deletion polymorphism and polyneuropathy in type 2 diabetes without macroalbuminuria. J. Int. Med. Res..

[43] Pavel J (2008). Expression and transport of Angiotensin II AT1 receptors in spinal cord, dorsal root ganglia and sciatic nerve of the rat. Brain Res..

[44] Mirzahosseini G, Ismael S, Ahmed HA, Ishrat T (2021). Manifestation of renin angiotensin system modulation in traumatic brain injury. Metab. Brain Dis..

[45] Pang L (2020). Understanding diabetic neuropathy: Focus on oxidative stress. Oxid. Med. Cell Longev..

[46] Alkhudhayri S (2021). Investigating the beneficial effect of aliskiren in attenuating neuropathic pain in diabetic Sprague-Dawley rats. Endocrinol. Diabetes Metab..

[47] Pabbidi RM, Cao DS, Parihar A, Pauza ME, Premkumar LS (2008). Direct role of streptozotocin in inducing thermal hyperalgesia by enhanced expression of transient receptor potential vanilloid 1 in sensory neurons. Mol. Pharmacol..

[48] Andersson DA (2015). Streptozotocin stimulates the ion channel TRPA1 directly: Involvement of peroxynitrite. J. Biol. Chem..

