# Supplementary material for: Clinical and preclinical evidence that angiotensin-converting enzyme inhibitors and angiotensin receptor blockers prevent diabetic peripheral neuropathy
Source: Sci Rep. 2024 Jan 10;14:1039. doi: 10.1038/s41598-024-51572-z (PMC10781693; doi:10.1038/s41598-024-51572-z)
Supplement: Supplementary file 1 — Supplementary Information. [file 41598_2024_51572_MOESM1_ESM.pdf]

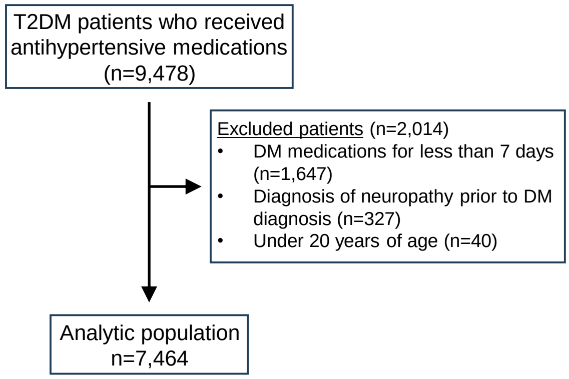

Supplementary Figure 1. Patient selection chart.

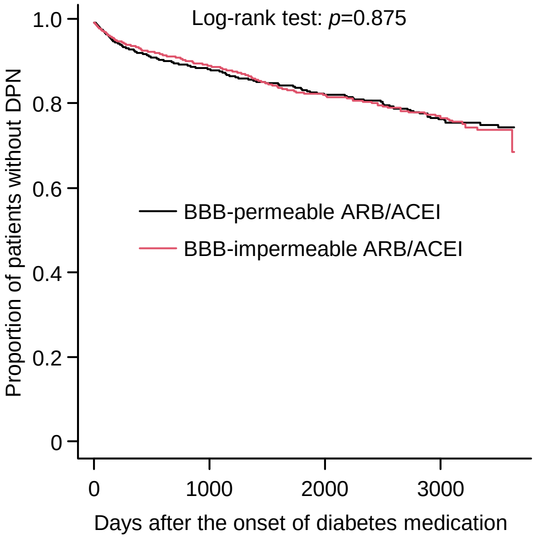

BBB-permeable	2032	831	482	213
BBB-impermeable	2927	1065	566	226

No. of residual patients

Supplementary Figure 2. Kaplan-Meier curves for the development of DPN in T2DM patients receiving BBB-permeable and BBB-impermeable ARB/ACEI**.** The patients receiving ARB or ACEI were divided into two groups according to BBB-permeability of the prescribed ARB/ACEI, i.e. BBB-permeable group (candesartan, valsartan, telmisartan, azilsartan, captopril, perindopril, lisinopril, temocapril) and BBB-impermeable group (losartan, irbesartan, olmesartan, enalapril, quinapril, imidapril).

Variables	Univariate analysis		Multivariate analysis	
	Hazard ratio (95% CI)	*p* value	Hazard ratio (95% CI)	*p* value
ARB or ACEI	0.61 (0.52-0.71)	**<0.001**	0.52 (0.39-0.69)	**<0.001**
CaB	1.09 (0.93-1.29)	0.29	0.99 (0.73-1.34)	0.94
History of smoking	0.89 (0.72-1.1)	0.27	0.81 (0.61-1.07)	0.14
High-LDL cholesterol	1.19 (0.87-1.62)	0.28	0.97 (0.53-1.80)	0.93
High-TG	1.02 (0.86-1.22)	0.80	1.06 (0.78-1.43)	0.71
Low-HDL cholesterol	1.29 (1.06-1.58)	**0.011**	1.37 (0.97-1.92)	0.073
Systolic blood pressure (mmHg)	1.00 (0.99-1)	0.16	0.82 (0.58-1.15)	0.25
Diastolic blood pressure (mmHg)	1.01 (1-1.02)	**0.007**	1.39 (0.78-2.47)	0.26

Supplementary Table 1. Sub-analyses of the association of variables including prescribed antihypertensive agents, smoking history, and the latest serum lipid profile and blood pressure values during the observation periods with DPN development in Kansai Medical University Hospital and Kindai University Nara Hospital, using univariate and multivariate cox hazards models. CI, confidence interval. ARB, angiotensin receptor blocker; ACEI, angiotensin-converting enzyme inhibitor; CaB, L-type calcium channel blocker; LDL, low density lipoprotein; HDL, high density lipoprotein; TG, triglyceride. The latest serum lipid profile, i.e. high-LDL cholesterol (≥140 mg/dL), high-TG (≥150 mg/dL) and low-HDL cholesterol (<40 mg/dL), and systolic and diastolic blood pressure values during the observation periods were used for the statistical analyses.
